# Mean platelet volume is associated with myocardial perfusion defect in diabetic patients

**DOI:** 10.5830/CVJA-2014-013

**Published:** 2014-06

**Authors:** Savas Sarikaya, Ali Riza Erbay, Safak Sahin, Lutfi Akyol, Elif Borekci, Yunus Keser Yilmaz, Fatih Altunkas, Kayihan Karaman, Seyhan Karacavus

**Affiliations:** Department of Cardiology, School of Medicine, Bozok University, Yozgat, Turkey; Department of Cardiology, School of Medicine, Bozok University, Yozgat, Turkey; Department of Internal Medicine, School of Medicine, Gaziosmanpaşa University, Tokat, Turkey; Department of Internal Medicine, School of Medicine, Bozok University, Yozgat, Turkey; Department of Internal Medicine, School of Medicine, Bozok University, Yozgat, Turkey; Department of Cardiovascular Surgery, School of Medicine, Bozok University, Yozgat, Turkey; Department of Cardiology, School of Medicine, Gaziosmanpaşa University, Tokat, Turkey; Department of Cardiology, School of Medicine, Gaziosmanpaşa University, Tokat, Turkey; Department of Nuclear Medicine, School of Medicine, Bozok University, Yozgat, Turkey

**Keywords:** myocardial perfusion defect, mean platelet volume, diabetes mellitus

## Abstract

**Aim:**

Our aim was to evaluate whether there was a relationship between mean platelet volume and myocardial perfusion defect in diabetic patients using myocardial perfusion imaging.

**Method:**

Forty-four diabetic patients with myocardial perfusion defect (group 1) and 44 diabetic patients without myocardial perfusion defect (group 2), matched for age and gender, were retrospectively examined. Levels of mean platelet volume (MPV) in the two groups were assessed.

**Results:**

MPV was higher in group 1 than group 2 patients (8.76 ± 0.76 and 8.25 ± 0.78 fl), respectively, *p* = 0.003). Levels of glucose, triglycerides (TG), total cholesterol (TC), low-density lipoprotein (LDL) cholesterol, high-density lipoprotein (HDL) cholesterol, haemoglobin (Hb) and glycosylated haemoglobin (HbA_1c_), and body mass index (BMI) in the two groups were not statistically significantly different. Multivariate logistic regression analyses showed that MPV was the only variable independently associated with myocardial perfusion defects (OR: 2.401, 95% CI: 1.298–4.440, *p* = 0.013).

**Conclusion:**

This study showed that higher MPV was associated with myocardial perfusion defects. Higher MPV in diabetic patients was independently related to myocardial perfusion defects and may be an indicator of myocardial ischaemia.

## Abstract

Diabetes mellitus (DM) is considered a coronary artery risk equivalent.^1^ DM is associated with an increased risk of cardiovascular morbidity and mortality.^2^,^3^ DM may cause myocardial perfusion defects involving the main coronary artery and myocardial microvascular circulation. Myocardial perfusion imaging (MPI) is a useful non-invasive tool to determine whether there is a myocardial perfusion defect.^4^

Platelet volume is a marker of platelet activation and function and is measured as mean platelet volume (MPV).^5^ MPV has become a prognostic factor in coronary heart disease and may eventually be accepted as a parameter of platelet activity.^6^ MPV is emerging as a new risk factor for vascular complications of DM of which atherothrombosis plays a crucial role.^7^

However, to the best of our knowledge, there have been no reports in the literature to evaluate the relationship between MPV and myocardial perfusion defect using MPI in patients with diabetes. Our aim was to evaluate whether there was a relationship between myocardial perfusion defect using myocardial perfusion scintigraphy and MPV in selected diabetic patients.

## Methods

Eighty-eight patients with type 2 diabetes who had MPI between January and May 2013 in Bozok and Gaziosmanpaşa universities were retrospectively examined. Eighty-eight patients were enrolled in the study and divided into two groups, matched for age and gender: the myocardial perfusion defect group (group 1) and a group with no myocardial perfusion defect (group 2). Group 1 consisted of 44 subjects (14 men and 30 women, mean age: 61.75 ± 7.86 years). Group 2 consisted of 44 subjects (12 men and 32 women, mean age: 60.48 ± 9.28 years).

Patients with a history of myocardial infarction, unstable angina pectoris, cardiac surgery, angiographically proven coronary artery disease, endocrine disorder without diabetes, systemic inflammatory disease, rhythm disorder, any medication that could affect the MPV, suspicious scintigraphy results due to breast attenuation, and aperture and fixed (scar) perfusion defects were excluded.

The blood samples were withdrawn following a 12-hour fast. Glucose, creatinine and lipid profiles were determined using standard methods. For both groups, we measured the MPV from blood samples that were obtained following venipuncture. The blood was collected in tripotassium EDTA tubes. We analysed the blood samples using an automatic blood counter within one hour of drawing the blood.

The patients underwent a two-day stress/rest single-photonemission tomography and gated GSPECT study using adenosine with a standard weight-based infusion protocol (140 μg/kg/min). The six-minute adenosine infusion was begun and 740 MBq (20 mCi) of MIBI was injected after three minutes. After a 45-minute delay, a stress set of images was acquired.

At rest, before receiving technetium-99m methoxy isobutyl isonitrile (^99m^Tc-MIBI), the patients were given one to two tablets of sublingual nitroglycerin (0.4 mg), five minutes apart and they were injected with 740 MBq (20 mCi) of MIBI. A GSPECT study was performed 45 minutes later.

GSPECT data were acquired in the supine position with the double-head SPECT-γ camera equipped with a high-resolution low-energy collimator. The obtained data were projected as myocardial tomographic slices in short-axis, vertical long-axis and horizontal long-axis views. Electrocardiogram gating was applied to the cardiac cycle with eight frames per cardiac cycle. The myocardium was divided into 17 segments following the American Society of Nuclear Cardiology/American College of Cardiology/American Heart Association guidelines.^8^

GSPECT dates were processed and analysed using 4D-MSPECT software, which determines the extent and severity of left ventricular perfusion defect size and the extent of reversible (ischaemia) or fixed (scar) perfusion defects.^9^ The programme assigned a score of 0 to 4 to each segment based on activity level: 0 = normal, 1 = equivocal, 2 = moderate, 3 = severe reduction of radioisotope uptake, and 4 = absence of detectable tracer uptake. Abnormal perfusion, motion and thickening were defined as a score of ≥ 2.

The summed stress score (SSS), summed rest score (SRS), and summed difference score (SDS) were calculated based on the conventional 17-segment model. The summed difference score (SDS), indicating the extent of reversible perfusion defects, was obtained by calculating the differences between the SSS and SRS.

## Statistical analysis

Statistical analyses were performed using SPSS 18.0 software. Parametric values are given as mean ± standard deviation and non-parametric values as a percentage. To compare parametric continuous variables, the Student’s *t*-test was used; to compare non-parametric continuous variables, the Mann–Whitney *U*-test was used. Categorical data were compared by chi-square distribution. Stepwise multivariate logistic regression models were created to determine independent variables for myocardial perfusion defect. For multivariate regression, variables with a *p*-value < 0.1 in univariate analysis were selected. Two-tailed *p*-values < 0.05 were considered to indicate statistical significance.

## Results

**Fig. 1.**
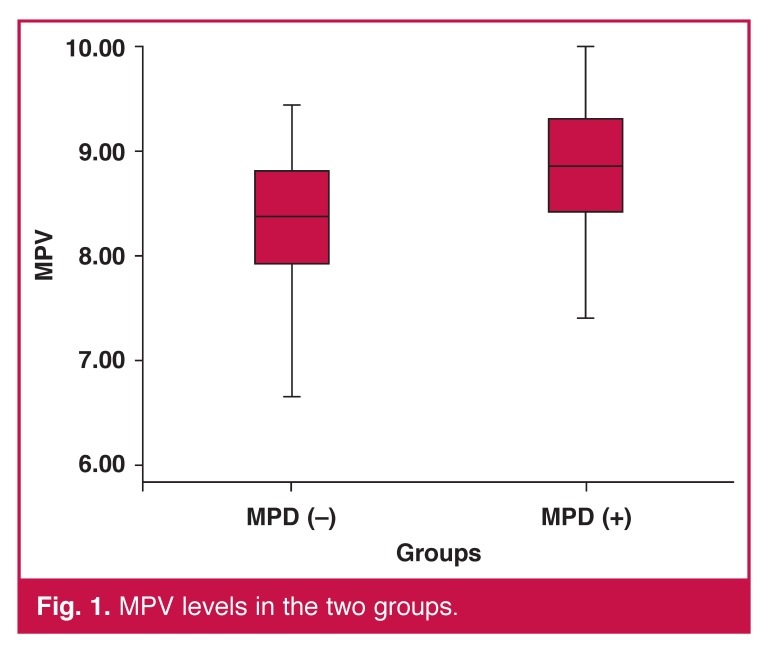
MPV levels in the two groups.

Baseline characteristic of the patients are given in Table 1. Levels of glucose, triglycerides (TG), total cholesterol (TC), low-density lipoprotein (LDL) cholesterol, high-density lipoprotein (HDL) cholesterol, haemoglobin (Hb) and glycosylated haemoglobin (HbA_1c_), and body mass index (BMI) in the two groups were not statistically significantly different. The MPV level was higher in group 1 than in group 2 patients (8.76 ± 0.78 and 8.25 ± 0.78 fl, respectively, *p* = 0.003). Levels of MPV in the two groups are shown in Fig. 1.

**Table 1:** Baseline characteristic of the patients.

	*Group 1*	*Group 2*	p*-value*
Age (years)	60.02 ± 9.28	60.81 ± 8.02	0.660
Women (%)	72.7	68.2	0.408
HT (%)	72.7	86.4	0.093
HL (%)	47.7	56.8	0.281
Aspirin (%)	34.1	29.5	0.410
BMI (kg/m^2^)	31.41 ± 6.23	30.41 ± 5.7	0.446
Glucose (mg/dl)	131.79 ± 40.553	151.16 ± 54.213	0.070
TG (mg/dl)	192.36 ± 116.48	171.71 ± 87.321	0.600
TC (mg/dl)	190.04 ± 42.25	178.83 ± 46.73	0.258
HDL-C (mg/dl)	40.58 ± 5.911	38.68 ± 6.08	0.167
LDL-C (mg/dl)	118.77 ± 28.75	108.28 ± 33.82	0.133
Hb (g/dl)	13.16 ± 1.40	13.42 ± 1.46	0.399
MPV (fl)	8.76 ± 0.76	8.25 ± 0.78	0.003
HbA_1c_ (%)	8.67 ± 0.68	8.35 ± 0.86	0.094

HT: hypertension; HL: hyperlipidaemia TG: triglycerides; TC: total cholesterol; HDL-C: high-density lipoprotein cholesterol; LDL-C: lowdensity lipoprotein cholesterol; Hb: haemoglobin; MPV: mean platelet volume; HbA1c: glycosylated haemoglobin.

Univariate analysis showed that MPV, and HbA_1c_ and glucose levels were significantly involved in myocardial perfusion defects. Multivariate logistic regression analyses showed that MPV was the only variable independently associated with myocardial perfusion defect (OR: 2.401, 95% CI: 1.298–4.440, *p* = 0.013) (Table 2).

**Table 2:** Univariate and multivariate regression analyses of independent variables for MPD .

*Variables*	*Univariate*			*Multivariate*		
	*OR*	*95% CI*	p*-value*	*OR*	*95% CI*	p*-value*
MPV (fl)	2.401	1.298–4.440	0.005	2.484	1.215–5.081	0.013
Glucose (mg/dl)	1.009	0.999–1.029	0.072	1.008	0.997–1.019	0.178
HbA_1c_ (%)	1.800	0.993–3.474	0.08	1.984	0.980–4.018	0.064
Age (years)	1.011	0.963–1.061	0.664			
Gender	1.244	0.497–3.16	0.641			
HT (mg/dl)	2.375	0.801–7.043	0.119			
BMI (km/m^2^)	0.991	0.92–1.067	0.820			
TC (mg/dl)	0.994	0.984–1.004	0.256			
TG (mg/dl)	0.998	0.994–1.002	0.360			
HDL-C (mg/dl)	0.948	0.878–1.023	0.167			
LDL (mg/dl)	0.989	0.975–1.003	0.134			
Hb (%)	1.138	0.845–1.534	0.395			

## Discussion

This study showed that there was a relationship between myocardial perfusion defect and MPV. MPV was higher in the group with myocardial perfusion defects, compared to the one without myocardial perfusion defects. Patients with diabetes develop vascular complications, including macrovascular complications [coronary artery disease (CAD), peripheral vascular disease and stroke] and microvascular complications [diabetic nephropathy (DN), diabetic retinopathy (DR) and peripheral neuropathy].^10^ Continuous hyperglycaemia may cause endothelial dysfunction and vascular lesions, resulting in diabetic vascular complications. 11,12

Type 2 diabetes is a substantial risk factor in atherosclerotic cardiovascular disease.^13^,^14^ Cardiovascular disease (CVD) is the leading cause of death in patients with type 2 DM.^15^ Asymptomatic CAD is common in patients with DM and is a strong predictor of future poor outcome of coronary vascular events, as well as early death.^16^,^17^ DM is associated with generalised endothelial dysfunction and small-vessel abnormalities.^18^,^19^

Perfusion defects are substantial predictors of coronary events in patients with known or suspected coronary heart disease (CHD).^20^ It is proposed that concomitant abnormalities of perfusion imaging scans in patients with diabetes with normal coronary angiograms may be caused by micro-angiopathy or endothelial dysfunction. Accordingly, it reflects an increased likelihood of future coronary events.^21^

The majority of studies on ischaemia have used SPECT MPI. An analysis of the diagnostic accuracy of pharmacologically induced stress MPI reported a mean sensitivity and specificity of 88 and 77%, respectively.^22^

Platelet volume is a marker of platelet activation and function, and is measured using MPV.5 Platelets that have dense granules are more active biochemically, functionally and metabolically. Large platelets secrete high levels of prothrombogenic thromboxane A2, serotonin, beta-thromboglobulin and procoagulant membrane proteins such as P-selectin and glycoprotein IIIa.^5^,^23^ Platelets secrete a large number of substances that are crucial mediators of coagulation, inflammation, thrombosis and atherosclerosis.^24^,^25^ It is also well known that large platelets are a risk factor for developing coronary thrombosis, leading to myocardial infarction.^19^,^23^,^26^,^27^

Measurement of platelet activation and/or aggregation may provide prognostic information in patients at risk for or following a cardiovascular event.^28^,^29^ Reports have revealed that there is a close relationship between MPV and cardiovascular risk factors, including impaired fasting glucose levels, diabetes mellitus, hypertension, hypercholesterolaemia, obesity and the metabolic syndrome.^30^-^32^ Increased platelet activity is reported to play a role in the development of vascular complications in diabetic patients.^18^

MPV was increased in patients with SCF complex and cardiac syndrome X, both being related to microvascular defects and endothelial dysfunction.^33^,^34^ In the present study, we showed that MPV was associated with myocardial perfusion defect, using MPI in diabetic patients.

In our study, MPV was increased in the myocardial perfusion defect group compared to those without myocardial perfusion defects. DM not only involves the main coronary artery but also the microvascular circulation, leading to myocardial perfusion defects. Perfusion defects are significant predictors of coronary events in patients with known or suspected CHD.^20^

The main limitation of our study was the small sample size, which could result in low statistical power for equivalency testing, leading to false-negative results. Second, because of the retrospective nature of data collection, the angiographic results of the patients were not evaluated. MPI may reflect myocardial perfusion defects but it was not able to show the anatomical status of the coronary artery. We cannot extend our results to the general population due to our broad exclusion criteria.

## Conclusion

MPV levels were higher in the diabetic patients with myocardial perfusion defects than in those without myocardial perfusion defects. In diabetic patients, increased MPV may be an independent marker of myocardial perfusion defects, which are associated with adverse coronary events.
